# Identification of candidate genetic variants and altered protein expression in neural stem and mature neural cells support altered microtubule function to be an essential component in bipolar disorder

**DOI:** 10.1038/s41398-020-01056-1

**Published:** 2020-11-09

**Authors:** Katarina Truvé, Toshima Z. Parris, Dzeneta Vizlin-Hodzic, Susanne Salmela, Evelin Berger, Hans Ågren, Keiko Funa

**Affiliations:** 1 Bioinformatics Core Facility, Sahlgrenska Academy at University of Gothenburg, Box 413, SE 405 30 Gothenburg, Sweden; 2 Department of Oncology, Institute of Clinical Sciences, Sahlgrenska Cancer Center, Sahlgrenska Academy at University of Gothenburg, Gothenburg, Sweden; 3 Institute of Neuroscience and Physiology, Department of Physiology, Sahlgrenska Academy at University of Gothenburg, Gothenburg, Sweden; 4 Institute of Neuroscience and Physiology, Department of Psychiatry and Neurochemistry, Sahlgrenska Academy at University of Gothenburg, Gothenburg, Sweden; 5 Proteomics Core Facility, Sahlgrenska Academy at University of Gothenburg, Gothenburg, Sweden; 6 Sahlgrenska Cancer Center at the Sahlgrenska Academy, Institute of Biomedicine, University of Gothenburg, Gothenburg, Sweden

**Keywords:** Bipolar disorder, Diagnostic markers

## Abstract

Identification of causative genetic variants leading to the development of bipolar disorder (BD) could result in genetic tests that would facilitate diagnosis. A better understanding of affected genes and pathways is also necessary for targeting of genes that may improve treatment strategies. To date several susceptibility genes have been reported from genome-wide association studies (GWAS), but little is known about specific variants that affect disease development. Here, we performed quantitative proteomics and whole-genome sequencing (WGS). Quantitative proteomics revealed NLRP2 as the most significantly up-regulated protein in neural stem cells and mature neural cells obtained from BD-patient cell samples. These results are in concordance with our previously published transcriptome analysis. Furthermore, the levels of FEZ2 and CADM2 proteins were also significantly differentially expressed in BD compared to control derived cells. The levels of FEZ2 were significantly downregulated in neural stem cells (NSC) while CADM2 was significantly up-regulated in mature neuronal cell culture. Promising novel candidate mutations were identified in the *ANK3, NEK3, NEK7, TUBB, ANKRD1*, and *BRD2* genes. A literature search of candidate variants and deregulated proteins revealed that there are several connections to microtubule function for the molecules putatively involved. Microtubule function in neurons is critical for axon structure and axonal transport. A functional dynamic microtubule is also needed for an advocate response to cellular and environmental stress. If microtubule dynamics is compromised by mutations, it could be followed by deregulated expression forming a possible explanation for the inherited vulnerability to stressful life events that have been proposed to trigger mood episodes in BD patients.

## Introduction

Bipolar disorder (BD) is a severe chronic psychiatric disorder, affecting >1% of the population worldwide^1^. The disease is characterized by recurrent episodes of mania and depression. About 15% of BD patients are expected to die from suicide^2,3^. Thus, early detection, diagnosis, and initiation of correct treatment are critical. The disorder has been shown to be largely genetically heritable, with estimates as high as 93% in twin-studies^4^. Genome-wide association studies (GWAS) have identified several risk loci, but these loci account for only a small portion of the heritability of BD^5^. The highly polygenic architecture of BD^6^ makes identification of causative variants challenging. Despite the complexity of the disease, we hypothesized that because of its high heritability it might be possible to identify common mis-regulated genes or pathways during neural development in BD. In a previous study, we integrated induced pluripotent stem cell (iPSC) technology^7^ with RNA-seq to investigate differences in the global transcriptome between BD patients and healthy controls^8^ using iPSC and neural stem cells (NSC)^9^. We found the *NLRP2* gene to be the most significant differentially expressed gene with a clear difference in expression for all cases and controls.

The *NLRP2* gene is a member of the nucleotide-binding and leucine-rich repeat receptor (NLR) family. Members of this family are thought to be important regulators of immune responses. Interestingly, *post-mortem* frontal cortex obtained from BD patients has been reported to show a high amount of a protein closely related to NLRP2, namely NLRP3^10^, in comparison to bio-samples from healthy individuals.

Although several susceptibility genes have been reported, no specific mutations have been shown to have any effect on disease development. Associated SNPs identified in GWAS are very rarely causative, but merely suggest that a causative mutation can be located close to the associated variant. In contrast, performing whole-genome sequencing (WGS) provides an opportunity to detect rare functionally relevant variants.

In the current investigation, we aimed to integrate results from our previous transcriptomics analysis with proteomics and whole-genome sequencing analysis. Since BD is a complex and highly polygenic disease, we expected to identify a few different mutations putatively contributing to the expression patterns observed in BD. We analyzed the difference in protein expression between BD patients and controls in both NSCs and mature neural cells. To explore the spectrum of variants within individuals, we performed WGS in cases with BD and healthy controls.

The focus of our research was to identify variants and genes that might be involved in the development of BD, with the ultimate goal of gaining knowledge that could lead to the development of diagnostic tests and improve current treatments.

## Results

### Generation of human iPSC, human iPSC-NSC, and human iPSC-neuron/glial cultures

**Fig. 1:**
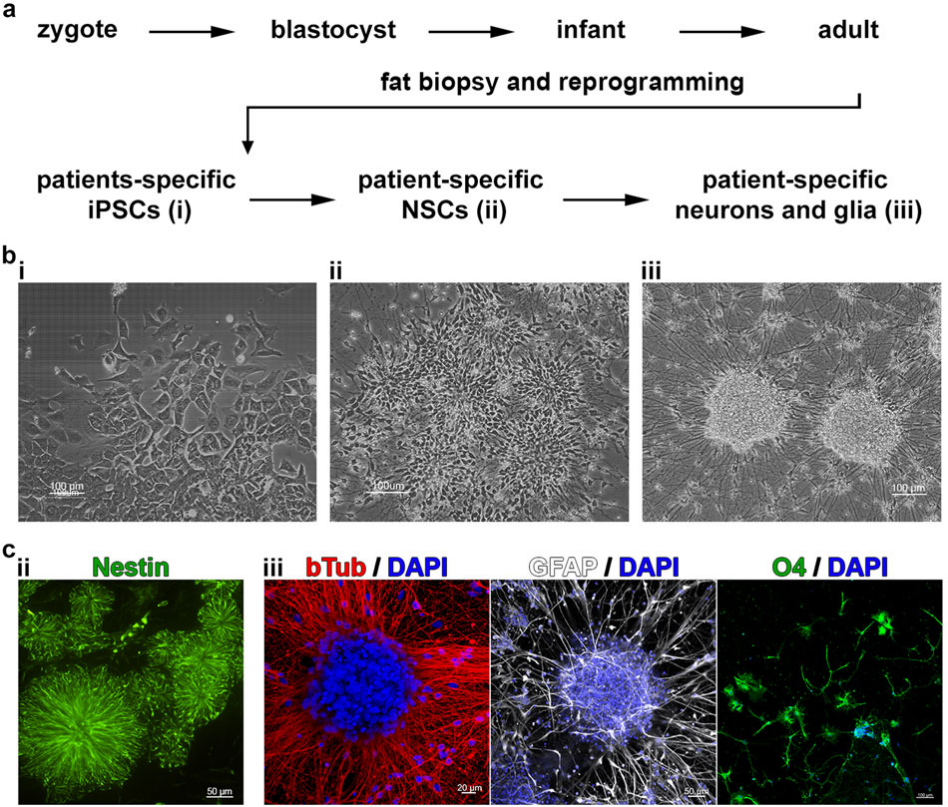
In vitro modeling of BD using iPSC technology and neural differentiation. **a** Schematic illustration of the major steps of human in vivo development and the application iPSC technology using adult somatic cells in order to generate BD-patient-specific neural cell cultures: i, ii, and iii stand for the different developmental stages (iPSC, NSC, and neuron/glial cells). **b** (i) Phase-contrast images show morphology of human iPSCs, (ii) hiPSC-derived NSC growing as neural rosettes, and (iii) generate 3D neural aggregates. **c** Fluorescent imaging visualizes (ii) Nestin^+^-NSC growing as neural rosettes 5 DIVs and (iii) beta-tubulin III^+^—neurons (left), GFAP^+^—astrocytes (middle) and O4^+^-oligodendroglial cells (right) in mature 3D neural aggregate cultures after 14 DIVs.

As described in our previous studies concerning the same sample^8,11^, abdominal subcutaneous adipose tissue was isolated and primary adipocyte cell lines were established and further reprogrammed into iPSC lines. A copy of the detailed method description from Vizlin-Hodzic et al.^8^ can be found in the supplementary information. In the present study, five BD-iPSC lines and four control-iPSC lines were used for the generation of NSCs and mature 3D neural aggregates comprising of neurons, astrocytes, and oligodendroglia cells^12^ (Fig. 1).

### Quantitative proteomics

**Fig. 2:**
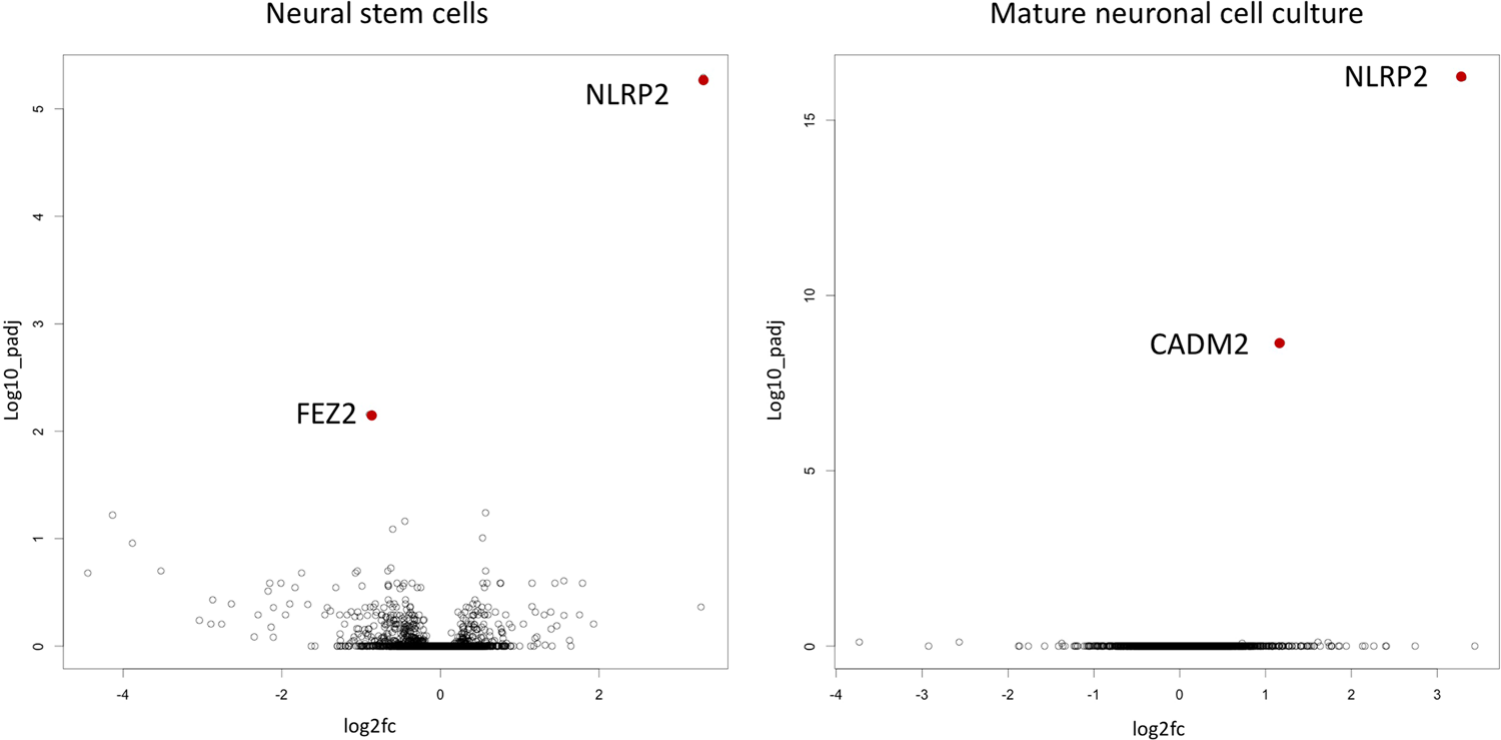
Volcano plots of protein expression. The volcano plot shows how the expression of proteins is distributed in the space of log2 fold change (log2fc) and adjusted *p*-values (log10_padj). Protein expression was investigated in neural stem cell and mature 3d neural aggregate cultures. Each dot represents the differential expression of one protein. Two proteins reached statistical significance between control and BD samples in each set after correcting for multiple testing.

**Fig. 3:**
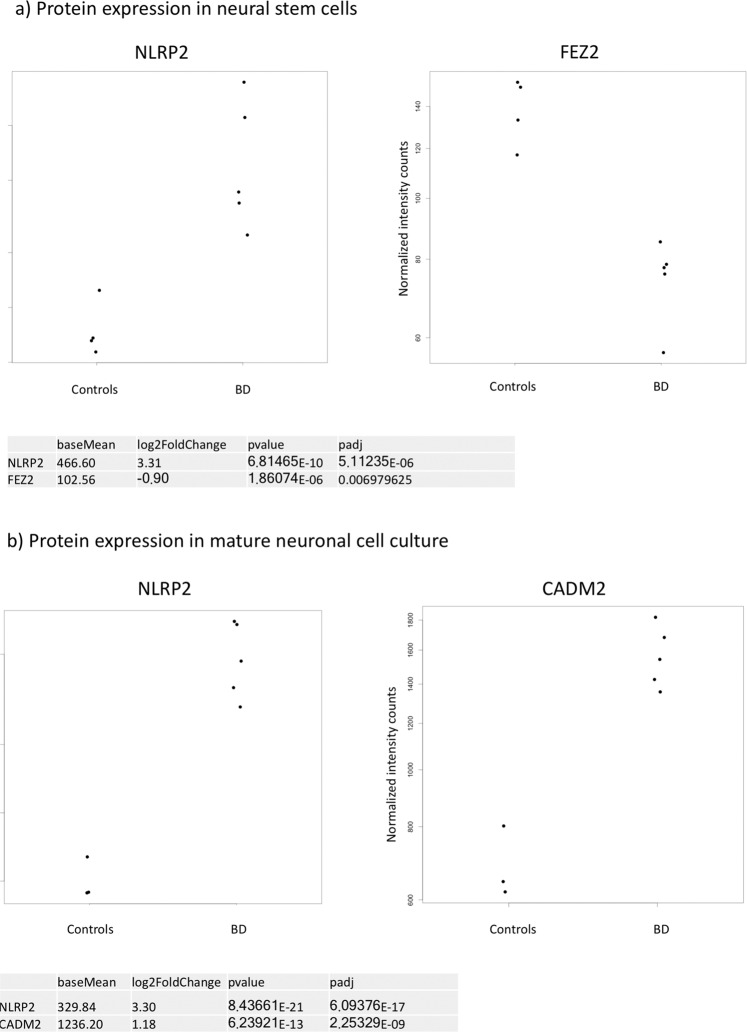
Protein expression per individual. Diagrams illustrate the normalized protein intensity counts for NLRP2 and FEZ2 in NSC cultures (**a**) and for NLRP2 and CADM2 in mature 3D neural aggregate cultures (**b**) obtained from bipolar disorder patient (BD) and healthy control iPSCs. Note, that the NLPR2, FEZ2, and CADM2 proteins that showed significant differences between BD and control groups in expression in (**a**) neural stem cells (3 DIVs) and (**b**) mature 3D neural aggregate cultures (21 DIVs). Each dot represents one individual.

We performed quantitative proteomics using tandem mass tags (TMT) to quantify protein levels in NSCs and mature 3D neural aggregate cultures obtained from five BD and four control-iPSC cell lines. For this purpose, we cultured BD and control NSC for 5 days, prior protein sampling preparation. In line with our transcriptome analyses^8^, NACHT, LRR, and PYD-containing protein 2 (NLRP2) were most significantly up-regulated in NSCs obtained from BD-patient cells (Fig. 2). In addition, the second most significant change in protein level in BD-NSC was fasciculation and elongation protein zeta-2 (FEZ2) (Fig. 2). In order to assess the protein levels in mature BD and control neural cells, NSCs were cultured for 14 days under neural differentiation conditions prior to protein sampling preparation. Even at this developmental stage, NLRP2 was identified to be the most significantly up-regulated protein in BD neural cell cultures in comparison to control lines (Fig. 2). The adhesion molecule 2 (CADM2) was the second most significantly up-regulated protein in BD-3D neural aggregate cultures (Fig. 2). In Fig. 3 the normalized protein intensities for each individual sample are plotted for the three gene products of *CADM2, FEZ2*, and *NLRP2*.

### Whole-genome sequencing

**Fig. 4:**
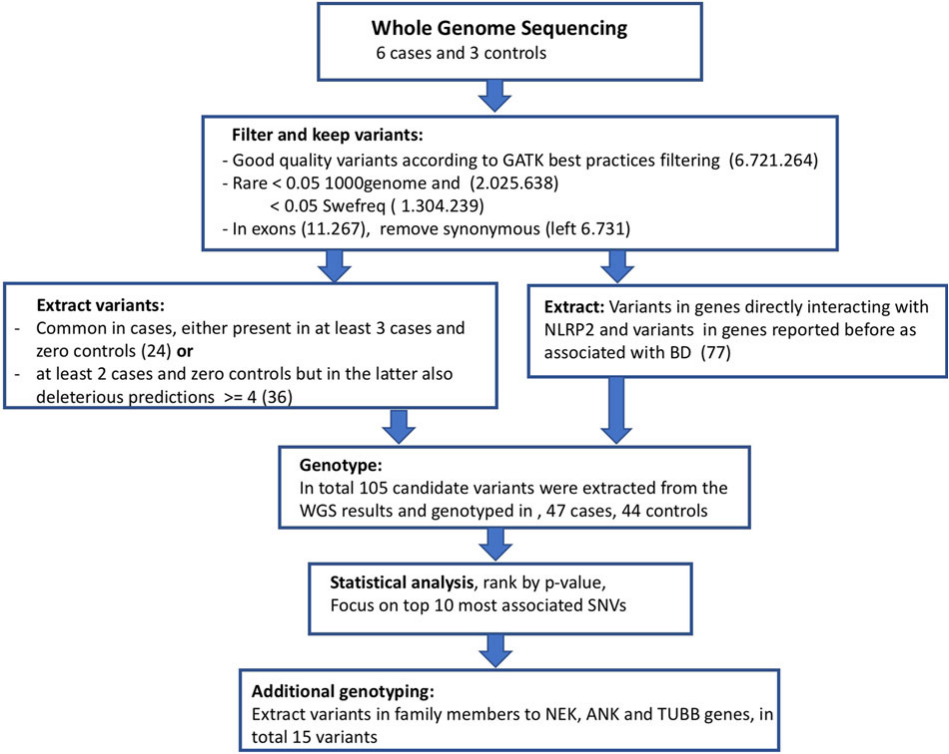
Filtering of WGS variants to be selected for genotyping in a larger cohort. First variants were filtered to only keep variants with good quality. Second variants were filtered to only keep variants, that are rare in a normal population. We kept variants that had an allele frequency lower than 5% in the 1000 Genomes^65^ and SweGen^56^ databases. Third, we decided to focus on exonic variants that cause a change in the amino acid sequence since these are most likely to affect protein function. Then, we wanted to prioritize variants that were seen in more than one case. In addition, we used several tools (see methods section for further details) that predict the effect of a variant at a certain position. The list of potential variants was sorted so that we first chose all variants seen in at least three cases and in no controls. Then, we selected the variants that were predicted to be deleterious by at least four tools, and that were present in at least two samples but in no controls. To this list, we extracted nonsynonymous variants that were identified in the following genes: *ANK3, BDNF-AS, C11orf80, CAGNA1C, CACNB3, COMT, DHH, HSP4, ITIH4, SYNE1, TENM4*, and *BRD2*. These genes were selected because of previously published reports suggesting relevance to BD or other psychiatric diseases. Since we hoped to explain the increased expression of *NLRP2* seen in RNA-seq^8^, we used the Biogrid (https://thebiogrid.org) database for protein-protein interactions and finally extracted all nonsynonymous variants in genes where the corresponding protein had been reported to physically interact with NLRP2. In each step, the number of selected variants can be found in parentheses.

To identify candidate mutations, WGS was performed using blood samples from unrelated patients (*n* = 6) and healthy controls (*n* = 3). These included the same subjects as were used for quantitative proteomics and the samples profiled with RNA-seq in our previous work for the purpose of identifying differentially expressed (DE) genes in NSCs^8^. Our aim was to extract for further investigation those variants most likely involved in disease development, and for this reason we used several criteria. We focused on variants that are rare in the general population since these are considered more likely to cause disease^13^. A schematic overview of filtering steps is presented in Fig. 4. From the WGS results we extracted a list of 105 candidate variants suitable for multiplex genotyping.

### Genotyping

The selected 105 SNPs were genotyped using the Sequenom MassARRAY iPLEX platform, where 84/105 variants (S1 Table) were informative and successfully genotyped in unrelated individuals (47 cases and 44 controls) (S2 Table). For the top 10 most associated variants, the allele frequency was checked in an additional dbGAP dataset (phs0008666.v1.p1; Table 1).

**Table 1:** Genotyping results.

Gene	Chr	Pos	F_A	F_U	P	F_1000 g	Del_Predict.	dbGAP
*First run of genotyping, top ten variants*								
NEK3	13	52707832	0.05319	0	0.02824	0.000798722	4	0/0
CSRP2BP	20	18162481	0.07447	0.01136	0.03796	0.0133786	4	6/5
ERC2	3	56330401	0.117	0.03659	0.04916	0.0141773	2	4/4
TRABD2A	2	85097590	0.06383	0.01136	0.06587	0.0245607	4	3/4
BRD2	6	32942355	0.06383	0.01136	0.06587	0.0109824	3	4/1
BRD2	6	32942354	0.06522	0.0119	0.07065	0.0109824	2	4/1
ANK3	10	61836174	0.03191	0	0.09106	0.000798722	6	0/0
TUBB1	20	57599557	0.03191	0	0.09106	0.00119808	6	0/0
RPL3L	16	2003016	0.07447	0.02273	0.1076	0.00599042	4	6/5
ANK1	8	41543675	0.05319	0.01136	0.1143	0.00978435	4	0/4
*Second run of genotyping*								
NEK7	1	198222215	0.02128	0	0.1788	0.00399361	2	4/1
ANKRD1	10	92675322	0.03191	0	0.09868	0.000199681	4	0/0

The top 10 most associated SNPs showing allele-frequency in affected (F_A), allele-frequency in unaffected (F_U), uncorrected Chi-square *p*-value (P), allele-frequency in 1000genome (F_1000g), the number of tools that predict the variant as deleterious (Del_predict), and number of cases/controls carrying the variant in the dbGAP dataset (phs0008666.v1.p1).

Among the top 10 most associated variants, the rarest variants in 1000genome and the control cohort are found in the *NEK3*, *ANK3*, and *TUBB1* genes (Table 1). In fact, none of the controls in this cohort had any of these variants, suggesting a high penetrance provided these variants are true positives. The *ANK3* gene has been reported in several GWA studies^14–16^ to be associated with BD, but to the best of our knowledge no causative variant has been identified in this gene. Interestingly, the most associated variant was located in the *NEK3* gene found in 5/47 cases. This gene has, to the best of our knowledge, not previously been reported to be associated with BD. NEK3 is a protein kinase which influences neuronal morphogenesis and polarity through effects on microtubules^17^. Tubulin is the major constituent of the microtubules and *TUBB1* codes for the tubulin beta-1 chain. Given the relevant functions of these genes, we decided to do an additional genotyping of 14 variants identified in the *NEK* gene family and some tubulin and ankyrin repeat genes (S3 Table). The procedure to filter out variants for further investigation is illustrated in Fig. 4. Two variants, one in *NEK7* and another in *ANKRD1* were the most associated in this second run of genotyping and were thus added to the list of possible candidates in Table 1.

### Comparison with dbGAP family data

In order to investigate whether the candidate gene variants were present in other cohorts, we applied for access to a dbGaP dataset (phs0008666.v1.p1) consisting of six large families affected with BD. Whole-genome genotyping data were present for 66 individuals, as previously described by Ament et al.^18^.

We examined this dataset for any overlap with the previously selected candidate variants (Table 1). The candidate variants in *NEK3, ANK3, ANKRD1*, and *TUBB1* were not present in any of these families. The variant in *NEK7* at position chr1:198222215 was present in one family (dbGaP ID “1201007”) in four cases and in one control. The family with the *NEK7* variant consisted of seven cases and three controls. Family members of BD patients frequently suffered from depressive disorders other than BD^18^, but information about these diagnoses were not included in the dbGAP dataset. From previous description of the data by Ament et al.^18^, it seems likely that all three controls were affected with “other” depressive disorders, and one of the cases suffered from major depression (dbGaP ID “1201007”). The mutation in *BRD2* was present in two families and in total four cases and one control. Some of the candidate variants turned out to be equally common or more common in family members that had not been diagnosed with BD (Table 1).

The analysis is complicated since some unaffected family members would likely be carriers of a not fully penetrant causative mutation. It has also been shown that BD shares genetic risk with other psychiatric diseases like schizophrenia and major depressive disorder^19^, that might affect some of the family members, but information about such diagnosis was not available to our study. We chose to focus on the variants where we saw a difference in frequency between cases and controls. For *NEK7, ANK3, NEK3, BRD2, ANKRD1*, and *TUBB1*, we decided to check dbGaP for the presence of other possible deleterious variants within these genes. We selected amino acid-changing variants with an allele frequency <0.01 in 1000genome and a difference >1 between cases and controls. The results are summarized in Table 2. A more detailed summary can be found in S4 Table.

**Table 2:** Variants in candidate genes dbGaP.

*Annotation*	*Gene*	*Chr*	*Start*	*End*	*Ref*	*Alt*	*1000g*	*Del*.	*Fam1*	*Fam2*	*Fam4*	*Fam5*
*nonframeshift substitution*	BRD2	6	32942354	32942355	GC	TG	.	NA		2/1	2/0	
*nonsynonymous SNV*	ANK3	10	61832711	61832711	T	C	.	0				3/0
*nonsynonymous SNV*	ANK3	10	61829273	61829273	T	C	0.0003993	3	3/1			
*nonsynonymous SNV*	ANK3	10	61833684	61833684	C	T	0.0013977	3	5/2			
*nonsynonymous SNV*	NEK7	1	198222215	198222215	C	G	0.0039936	2			4/1	
*Total number of cases/controls*									6/4	3/7	7/3	7/1

Variants from selected candidate genes were checked in the dbGaP database (phs0008666.v1.p1). Number of cases/controls carrying the variant is reported for the families were these variants were present. The total number of cases and controls for each family is shown in the last row. Del. is short for the number of deleterious predictions for the variant.

We assume that the rarer the variant in the general population (e.g. 1000 g), and the more deleterious the predictions, the more likely it is to be a true positive. We cannot exclude any of the variants presented in Table 1 as possibly contributing to the disease, but we decided to focus on variants that are rare in controls both in the dbGaP family data and in the study of unrelated cases and controls. Integrating the results from genotyping of unrelated cases and controls (Table 1) and the presence of variants in candidate genes within the dbGaP family dataset (Tables 1, 2), we consider the variants presented in Table 3 as the most promising candidate variants.

**Table 3:** Selection of most promising candidates.

*Annotation*	*Gene*	*Chr*	*Start*	*End*	*Ref*	*Alt*	*1000g*	*Del*.	*Genotyping*	*dbGaP*
*nonframeshift substitution*	BRD2	6	32942354	32942355	GC	TG	.	NA	6/1	4/1
*nonsynonymous SNV*	ANK3	10	61832711	61832711	T	C	.	0		3/0
*nonsynonymous SNV*	ANK3	10	61829273	61829273	T	C	0.0003993	3		3/1
*nonsynonymous SNV*	ANK3	10	61833684	61833684	C	T	0.0013977	3		5/2
*nonsynonymous SNV*	ANK3	10	61836174	61836174			0.00079872	6	3/0	
*nonsynonymous SNV*	NEK7	1	198222215	198222215	C	G	0.0039936	2	2/0	4/1
*nonsynonymous SNV*	NEK3	13	52707832	52707832	A	G	0.00079872	4	5/0	
*nonsynonymous SNV*	TUBB1	20	57599557		A	G	0.00119808	6	3/0	
*nonsynonymous SNV*	ANKRD1	10	92675322	92675322	A	G	0.00019968	4	3/0	
*Total number cases / controls*									47/44	30/36

Integration of rare variants from genotyping in unrelated cases and controls (column Genotyping) with variants in selected candidate genes that showed a difference between cases and controls in the dbGaP family dataset (phs0008666.v1.p1). Number of cases/controls carrying the variant is reported for each variant. Last row shows the total number of cases and controls present in the two datasets. Del. is short for number of deleterious predictions for the variant.

## Discussion

The aim of this study was to identify genes, proteins, and genetic variants involved in the development of BD. Our sample size was relatively small, and the nature of the disease is complex. Nevertheless, we figured that even with a small sample it would be possible to identify a few variants with a putative effect on disease development, focusing on filtering out the most likely variants, and using several sources of information to evaluate the identified variants.

### Differentially expressed proteins

Using quantitative proteomics, we found that the NLRP2 protein was up-regulated in BD-patient cells and the most significantly differentially expressed protein comparing NSC and mature neural progeny from BD patients and controls cells. This was in concordance with our previous results using RNA-seq data measured at the transcript level^8^. Secondly, we identified downregulation of fasciculation and elongation protein zeta-2 (FEZ2). *FEZ2* gene expression has previously been shown to be induced in rats treated with methamphetamine as an animal model for psychotic mania^20^. We showed that, when NSCs had differentiated into mature neurons and glial cells, NLRP2 was still the most significantly differentially expressed protein, followed by a significantly up-regulated adhesion molecule 2 (CADM2). CADM2 is a brain-specific protein that functions as a cell adhesion molecule at the synapse. Expression of its cytoplasmic tail in neurons has been shown to inhibit synapse assembly while expression of full-length CADM2 in non-neural cells induces synapse assembly^21^. Furthermore, CADM2 has been reported in a GWAS study to be associated with risk-taking behavior, which is a key component that shares a significant genetic risk with several psychiatric disorders including BD^22^.

### Why integrate expression analysis with WGS

Deregulated expression could be caused by mutations in genes with whom the deregulated genes/proteins interact either directly or indirectly, i.e. the effect might be visible after a chain of reactions with several interactors involved. Since BD patients share a similar phenotype but display a high level of genetic heterogeneity, it could be assumed that this similarity in phenotype could be explained by different mutations in the same gene, or by mutations in different genes involved in the same cellular task or the same signaling network.

### WGS in comparison to GWAS

By performing WGS followed by genotyping of candidates in a larger cohort, we identified several novel candidate variants that may affect the development of BD. We emphasize that in this study we focused on rare nonsynonymous variants, since these are the most likely to affect protein function. We also note the difference between a GWA study and WGS. In a GWAS, a number of marker SNPs are used and results rely on linkage disequilibrium, meaning that it utilizes the fact that close variants in the genome are inherited together. Variants, although significantly associated in a GWA study may not be causative, but more likely to be close to genomic loci harboring a causative variant. The majority of reported SNPs from GWA studies are located in introns^23^ and are thus less likely to be causative, even though some intronic variants may have a regulatory function. In the case when there are several different rare causative mutations in the same gene, having arisen during different events and affecting different subgroups of the population, the surrounding marker SNPs will probably not be very informative, resulting in false negative genes. Use of WGS is more expensive than the use of SNP-arrays and sequencing projects are most often run with sample sizes that are too small to produce any significant results at the genome level. On the other hand, sequencing offers direct information about alleles at all positions and will allow detection of rare variants when the surrounding regions are not informative.

### Novel ANK3 variants identified

A number of the variants identified in this study were located in the *ANK3* gene. This gene has been reported to be associated with BD in several GWA studies^23,24^, but most of the specific variants identified here have, to our knowledge, not been previously reported. In this study, we found an *ANK3* exonic variant at position chr10:61836174 in 3/47 cases in our dataset. In the dbGaP cohort we identified five other *ANK3* exonic variants to be present in 8/30 cases, with most of the variants clustering in one family. We noted that the mutation located at position chr10:61832711 has been previously reported to be significantly associated with BD by Fiorentino et al.^25^. The other variants have, to our knowledge, not been reported as implicated in BD development. Given these numbers (≈26% of cases in the dbGAP dataset) carrying rare nonsynonymous variants in *ANK3*, it seems possible that *ANK3* mutations explain a large fraction of BD cases. This would contrast to what has previously been estimated where an almost zero proportion of SNPs would account for a large effect (each SNP 1% of the variance) in BD^6^, when estimations were based on SNP markers.

Ankyrins are adaptor proteins that form protein complexes consisting of ion channels and transporters, cell adhesion molecules, signaling proteins, and cytoskeletal elements. Regulation of neuronal excitability is a developmental process where basket interneurons form GABAergic synapses with axon initial segments (AIS) of Purkinje neurons^14^. Since this process is dependent on ANK3 function, the proposition that a deficiency in this gene could lead to BD is consistent with the hypothesis that BD is related to changes in synaptic connectivity^14,15^.

### NIMA-related kinases and a possible link to NLRP2 expression

*ANK3* mutations appear to be promising candidates, but they will not explain all BD cases. In our study, NIMA-related kinases (Nek) emerged as another group of promising candidate genes. To our knowledge, Nek genes have not been suggested to be involved in BD, although the genetic region containing two gene family members, *NEK4* and *NEK7*, has been reported to be associated in large meta GWA study^26^. In our study, we identified a candidate in *NEK3* carried by 5/47 BD cases at position chr13:52707832 and one candidate in *NEK7* at position chr1:198222215 carried by 2/47 BD cases in our dataset. The same *NEK7* variant was found in four cases of one family in the dbGaP dataset. Gene functions for this gene family are likely to be relevant for BD. Some NIMA family members have been shown to be involved in cilium formation and length^27^. *NEK3* has been shown to affect neuronal microtubule acetylation^17^. The microtubule dynamic instability has been shown to be dependent of *NEK7*^28^. Furthermore, it has been shown that NEK7 is essential for the activation of the NLRP3 inflammasome^29^. This would suggest that members of the NEK family also affect the expression of the closely related NLRP2 which warrants further investigation.

### Microtubule function and its connection to stress

Molecular networks are complex, and it can be difficult to evaluate the significance of functional similarity. The candidate genes we selected were based on an observed difference between cases and controls, but a literature study revealed that there are also striking connections to microtubule function for several of the candidate genes, especially *ANK3*, *NEK3*, *NEK7*, and *TUBB1*. In axons, microtubules are responsible for maintaining axon structure and axonal transport^30^. Dendritic microtubules influence processes like arborization and signaling to dendritic spines^31^. TUBB1 is a component of the microtubule that has been proposed to be present in the postsynaptic density of dendritic spines^32^. A direct protein interaction has been shown between microtubule end-binding proteins EB1/EB3 and ANK3^33^, and disruption of *ANK3* has been shown to increase microtubule dynamics^34^. NEK3 and NEK7 are involved in microtubule acetylation and stability^17,28^. It has been noted before that there is a connection between a dysfunctional microtubule cytoskeleton, disrupted synaptic, and neuropsychiatric illness^31^. Furthermore, stressful life events often trigger mood episodes in BD^35^, and neuronal plasticity is essential for an adaptive response to adverse situations e.g. exposure to stress^31^. Microtubules actively modify their structure through dynamic cycles of assembly and disassembly^36^. Posttranslational modification like acetylation and deacetylation of tubulin has been shown to be affected by exposure to stress and has also been proposed to be involved in the pathology of depression^31^. Deacetylation of tubulin is executed by HDAC6^37^ and is dependent on NEK3^17^. HDAC4 and HDAC6 have been reported to be differentially expressed in individuals with BD compared to controls^38^. There are also possible links with the differentially expressed protein FEZ2 and microtubule function. FEZ2 has been shown to colocalize with tubulin where it may play a role in autophagy, a process important for maintaining homeostasis during critical times such as in conditions of cellular or environmental stress conditions. It is also possible to find some indirect links between NLRP2 and tubulin. NLRP2 has been shown to physically bind to the protein CRIPT^39^ and CRIPT in turn has been shown to colocalize with tubulin in the postsynaptic density where it directly binds to microtubule^32^.

### Other candidates

Bromodomains (BRDs) bind to acetyl–lysine binding sites^40^ and it has been suggested that BRD2 can inhibit HDAC deacetylation^41^. Another bromodomain gene, *BRD1*, has been proposed as a susceptibility gene for both schizophrenia and BD^42^. There are, to the best of our knowledge, no reports of ANKRD1 function in the brain, but our previous work confirmed^8^, that *ANKRD1* was expressed at the transcript level in neural stem cells. *ANKRD1* has been reported to be induced by *ERK5* during neural differentiation in adrenal medullary cells, regulating TH levels and catecholamine biosynthesis^43^.

### Several functions for NLRP2

NLRP2 has been shown to be the most significantly differentially expressed gene/gene product in BD comparing both at the RNA and protein levels. NLRP2 has been reported to have different functions in different cell types. NLRP2 is expressed in oocytes where it has been suggested to regulate early embryo development^44^. In the brain, NLRP2 has been reported to be expressed in the astrocytes where it has been suggested to be an important component of the CNS inflammatory response^45^. It is not known what function NLRP2 has in neurons, but its interactions with CRIPT make us hypothesize that NLRP2 is localized to the postsynaptic density in neurons and related to microtubule function. Since NLRP2 has been reported to be part of an inflammatory response, deregulation may indicate involvement in the dysregulation of immuno-inflammatory pathways reported in BD patients^46^. Cytokines released by astrocytes during inflammatory response also play a role in the dysregulation of neural cell homeostasis^46^.

### Limitations

Given the polygenic nature of BD and the small sample size, we did not have enough statistical power to use standard cut-off p-values as a measure of significance when evaluating single polymorphic variants. Instead, we used statistics to rank variants, thus variants shared by several cases, and none or very few controls would be selected as the most interesting variants. The only gene/protein that was significantly differentially expressed at both RNA and protein level was NLRP2. It has been noted before that mRNA and protein levels are modestly correlated^47^. A possible explanation is that post transcriptional regulation has a strong effect on protein levels i.e. pathways for ubiquitination and proteasomal degradation.

## Conclusions

We have identified novel candidate mutations and proteins putatively involved in bipolar disease development. Genotyping larger samples could be helpful to investigate the allele frequency in BD of the variants identified here and of other variants in these candidate genes. It should be remembered that association alone never proves causality. For evaluation of function, it would be important to invest in molecular functional studies exploring the effect of these variants on protein function. It also remains to be investigated whether any of the identified novel candidate variants could explain the up-regulated expression of NLRP2, and how that would in turn lead to disease development. Ultimately, *NLRP2* or genes affecting its expression could be candidates for further investigation as targets for pharmacological treatment.

## Methods

### Sample selection

In total, 47 Caucasian patients from the Bipolar Outpatient Department at the Psychiatric Clinic, Sahlgrenska University Hospital (Gothenburg, Sweden) were enrolled in this study. Diagnoses were validated through structured psychiatric interviews i.e. Mini-International Neuropsychiatric Interview (M.I.N.I.), version 6^48^ in an authorized Swedish translation, yielding DSM-IV (Diagnostic and Statistical Manual of Mental Disorders, Fourth Edition) diagnoses. For controls, 44 healthy Caucasians were selected and interviewed in the same way as patients to validate their status as psychiatrically healthy. The Regional Research Ethics Board in Gothenburg approved the study (#172-08). Informed consent was obtained from all patients. Whole-genome sequencing was performed in six of these patients diagnosed with BD and three controls. The same cases and controls had previously been analyzed with RNA sequencing as described in Vizlin-Hodzic et al.^8^. All six initial cases were diagnosed with BD type I (i.e., patients had been manic and psychotic at some phase in their psychiatric history). We only sent three controls for genome sequencing, since we figured that sequencing of controls are not equally important for variant filtering, as they are in expression studies. They are important to be able to remove sequence artifacts but public databases such as 1000genome and SweGen were very helpful to filter variants.

Concerning quantitative proteomics, for one of these six BD patients, the cells did not grow, and for one control the cells did not grow into mature neuronal cell cultures and thus had to be discarded.

### Human iPSC, human iPSC-NSC, and human iPSC-neuron/glial cultures

All iPSC lines were cultured under feeder-free conditions in Cellartis DEF-CS™ (Takara Bio Europe AB) at 37 °C in a humidified atmosphere of 5% CO_2_ in air. Media exchanges were performed daily. Human iPSC was used for neural induction by applying the DUAL-SMAD inhibition protocol^8^. The detailed neural differentiation procedure for iPSC lines is described in supplementary information and elsewhere^8,11^. Cryostock of hiPSC-NSC cultures were generated by passaging cells using Accutase 20 to 24 days post neural induction in hiPSC cultures. Cell suspension of hiPSC-NSC was stored in 10% DMSO solution and cryostocks were kept at −152 °C. Frozen stocks of hiPSC-NSC were thawed and 1.0 × 10^6^ cells were cultured in neural culture media (NM) comprised of DMEM/F12 GlutaMAX, Neurobasal, N2 supplement, B27 supplement without vitamin A, 5 µg ml^−1^ insulin, 1 mM Ultra glutamine, 100 µM non-essential amino acids, 100 µM 2-mercaptoethanol, 50 U ml^-1^ penicillin and streptomycin (ThermoFisher) on biolaminin 521 [5 µg/ml] (BioLamina) coated 3.5 cm culture plates or coverslips for immunofluorescent staining. Media exchanges were performed every 2–3 days. For the generation of human iPSC-neuron/glial cultures, 3D neural aggregates were isolated from confluent hIPSC NSC cultures (7 days in vitro) and sub-plated on biolaminin 521-coated plates (for protein isolation) or glass coverslips (for immunofluorescent imaging). To enhance maturation of hiPSC-NSC into neurons and glial cells, 3D neural aggregates were cultured in BrainPhys-media supplemented with N2 supplement, B27 with vitamin A, 2 mM Ultra glutamine, 50 U ml^−1^ Pen/Strep, and 200 µM ascorbic acid were used. Half media exchanges were performed twice a week. Human BNDF, GNDF, NT-3, FGF8, TGF-β, (20 ng/ml each, all Peprotech) and DAPT (Tocris) were used as neurotrophic factors. For details about the generation and characterization of 3D neural aggregates see elsewhere^12^.

### Immunocytochemistry and confocal imaging

For immunocytochemistry, hiPSC-NSCs and neural aggregates were cultured on glass coverslips. The protocol for immunocytochemistry is described in our previous study^12^. Neural stem cell cultures were stained with mouse Nestin (Abcam, ab22035, 1:500) antibody, and 3D neural aggregate cultures with mouse betaTubIII (R&D, MAB1195, 1:2000) to visualize neurons, rabbit GFAP (DAKO, now Labome, Z0334, 1:1000) to visualize astrocytes and mouse O4 (Abcam, ab53041, 1:200) to visualize oligodendroglial cells. Secondary antibodies (goat-anti mouse-488, goat-anti rabbit-568 (ThermoFisher, all 1:500) were used for fluorescent labeling. Confocal imaging was performed with LSM 710 META (Zeiss).

### Quantitative proteomics

Confluent cultures of hiPSC-NSC (5 days in vitro) or differentiated 3D neural aggregate cultures (14 days in vitro) were washed with PBS and lysed by beat-beating in 50 mM triethylammonium bicarbonate (TEAB), 2% sodium dodecyl sulfate (SDS). Protein samples were prepared for proteomics analysis by filter-aided sample preparation. Shortly, proteins were reduced, alkylated, and trypsin-digested on 30 kDa cut-off filters. Resulting peptides were labeled using TMT 10-plex isobaric mass tagging reagents (Thermo Scientific), and pooled into two sets of samples (neural stem cells and mature neural cells, respectively). Subsequently, off-line separation was performed using basic reverse-phase chromatography, and the resulting 20 peptide fractions were subjected to nanoLC-MS/MS analysis. Raw files of all fractions per TMT set were merged, and proteins were identified and quantified using Proteome Discoverer (version 2.2, Thermo Fisher Scientific) by matching against the human SwissProt database (Jan 2019). A protein false-discovery rate of 1% was applied. A more detailed description is available in supplementary material. Differential protein abundance between the two sample groups was determined using the DESeq2 package^49^. Data normalization between samples and corrections for multiple testing was performed within the package. The use of DESeq package for proteomic spectral count quantitation has been proposed by Branson and Freitas^50^. The experiment was performed once.

### Whole-genome sequencing

DNA was extracted from blood samples using DNeasy Blood and Tissue kit (Qiagen).

Library preparation and sequencing were performed by the National Genomics Infrastructure in Stockholm validated under ISO accreditation 17025:2005. Library type was Illumina TruSeq PCR-free with a read length of 150 bp. Samples were sequenced on HiSeqX. The Bcl to FastQ conversion was performed using bcl2fastq-v2.17.1.14 from the CASAVA software suite. The quality scale used was Sanger/phred33/Illumina 1.8+.

Quality control of whole-genome sequencing data was performed using Qualimap^51^ and Fastqc (S1 Figure). Data were aligned to the reference (human hg19) using a Burrows Wheeler Aligner (BWA version 0.7.12)^52^. Samtools (version 0.1.19)^53^ was used to sort index and assess mapping statistics. The number of reads sequenced and the percentage mapped per individual is shown in S5 Table. The Genome Analysis Toolkit GATK (version 3.5)^54^ best practice was used for realignment and recalibration. SNVs and Indels were called using the GATK tool HaplotypeCaller. The following hard filter was applied for quality filtering: for SNPs: QD < 2.0, MQ < 40.0, FS > 60.0, ReadPosRankSum < −8.0, MQRankSum < −12.5, and for indels: QD < 2.0, FS > 200.0, ReadPosRankSum < −20.0. Called variants that passed the GATK quality filtering were further filtered against 1000 Genomes^55^ and SweGen^56^ to remove variants with an alternative allele frequency >0.05. Remaining variants were annotated with the knownGene (based on the UCSC genome browser knownGene track) database using the Annovar tool^57^. Annovar was further used to annotate exonic variants with functional predictions from the following tools: SIFT^58^, PolyPhen2-HDIV^59^, LRT^60^, MutationTaster^61^, MetaLR^62^, FATHMM^63^, and Radial-SVM^62^. For the dbGaP dataset (phs0008666.v1.p1) the data were downloaded as vcf-files and after that filtered and annotated in the same way as above. The experiment was performed once. Bam-files are available via controlled access at EGA dataset EGAD00001006082.

### Genotyping of filtered variants

DNA was extracted from blood samples using DNeasy Blood and Tissue kit (Qiagen). The genotyping was performed using a multiplexed primer extension (SBE) chemistry of the iPLEX assay with detection of the incorporated allele by mass spectrometry with a MassARRAY analyzer from Agena Bioscience. Raw data from the mass reader was converted to genotype data using the Typer software (Agena Bioscience). Reproducibility was controlled to be 100%. The tool PLINK (version 1.07)^64^ was used for Chi-square calculations to test for association between SNV allele and phenotype. The experiment was performed twice.

## Supplementary information

Neural differentiation procedure

Protein extraction, digestion and labeling

Figure S1

Table S1

Table S2

Table S3

Table S4

Table S5
