# Supplementary material for: Identification of candidate genetic variants and altered protein expression in neural stem and mature neural cells support altered microtubule function to be an essential component in bipolar disorder
Source: Transl Psychiatry. 2020 Nov 9;10:390. doi: 10.1038/s41398-020-01056-1 (PMC7652854; doi:10.1038/s41398-020-01056-1)
Supplement: Supplementary file 1 — Neural differentiation procedure [file 41398_2020_1056_MOESM1_ESM.docx]

**Description of neural differentiation procedure as earlier published in Vizlin-Hodzic et al., *Translational Psychiatry* 7**, e1010(2017)

### “Generation of hiPSC lines and culture conditions

### Abdominal subcutaneous adipose tissue was isolated and primary adipocyte cell lines were established (Hayashi et al., 2015).Starting with adipocytes from patients (B1, B2, B3, B4, B5, B6) and controls (C1, C2 and C3), iPSCs were generated and characterised by Cellectis (formerly Cellartis, presently Takara Clontech, Shiga, Japan). As an additional and study-independent control, one Cellartis DEF-hiPSC ChiPSC4 line (C4) was used. All lines were cultured under feeder-free conditions in Cellartis DEF-CS (Takara Bio Europe, Gothenburg, Sweden) at 37 °C in a humidified atmosphere of 5% CO_2_ in air. Primary adipocytes were cultured as described earlier (Hayashi et al., 2015) without supplement of insulin, dexamethasone or other reagents that maintain adipocyte characteristics.

To create an in vitro BD model system, adipocyte cell lines were thus originally derived from abdominal subcutaneous fat samples of altogether 35 BD (11 BD I, 7 BD II and 17 BD non ultra descriptus) patients and 38 healthy controls, as earlier described (Hayashi et al., 2015). From these cell lines, six BD I and three healthy controls were selected on technical grounds. The six BD I patients (three females, three males) donating abdominal adipocytes were all euthymic at the time of examination with GAF scores (±s.d.) 69.2±13.6, Young Mania Rating Scale 0.7±1.6 and Montgomery–Åsberg Depression Rating Scale 5.7±5.1. They all had suffered from at least one psychotic episode in their psychiatric history. All have Caucasian origins. Their age at first mood episode compared with their current age was 12/53, 18/39, 17/44, 15/47, 15/47 and 21/32 years. Four were currently medically mood-stabilised with lithium, four with antipsychotics and two with antiepileptics. Three controls were healthy females, age 27, 26 and 29 years. One ChiPSC4 line (C4) male control was added.

### Directed differentiation of hiPSC

The hiPSCs lines were plated on COAT1 (Takara Bio Europe) coated 12-well plates in feeder-free conditioned Cellartis DEF-CS medium (Takara Bio Europe) until confluence. Neural induction was initiated by changing the culture medium to 1:1 mixture of N2 media, consisting of DMEM/F12 GlutaMAX (Life Technologies, Carlsbad, CA, USA), N2 supplement (Life Technologies), 5 μg ml^−1^ insulin (Sigma-Aldrich, St. Louis, MO, USA), 1 mm Ultra glutamine (Lonza, Basel, Switzerland), 100 μm non-essential amino acids (Gibco, Carlsbad, CA, USA), 100 μm 2-mercaptoethanol (Gibco), 50 U ml^−1^ penicillin and streptomycin (Lonza), and B27 media (Neurobasal; Life Technologies), consisting of B27 with vitamin A (Life Technologies), 2 mm Ultra glutamine (Lonza), 50 U ml^−1^ penicillin and streptomycin (Lonza) supplemented with 1 μm Dorsomorphin (Tocris Bioscience, Bristol, UK) and 10 μmSB431542 (Tocris Bioscience). Neural induction media was replaced every day for 8–10 days until a uniform neuro-epithelial sheet was observed, and then both iPSC and neuro-epithelial cells were frozen in RNA protect (Qiagen, Redwood City, CA, USA) for miRNA and transcriptome analysis. Neuro-epithelial cells were collected by dissociation with Dispase (Life Technologies) and aggregates were re-plated on laminin-coated plates and maintained in neural maintenance media. On appearance of rosette structures, NSCs were expanded by supplementing media with 20 ng ml^−1^FGF2. After a further 4 days, FGF was withdrawn and the cultures were passaged using Accutase and maintained in a neural maintenance media until frozen at day 23–30 post initiation of neural induction. For neurogenesis, NSCs were cultured on poly-l-ornithine/laminin-coated dishes on a feeder layer of human astrocytes. Neural maintenance media was changed every second day.”
