# Supplementary material for: Identification of candidate genetic variants and altered protein expression in neural stem and mature neural cells support altered microtubule function to be an essential component in bipolar disorder
Source: Transl Psychiatry. 2020 Nov 9;10:390. doi: 10.1038/s41398-020-01056-1 (PMC7652854; doi:10.1038/s41398-020-01056-1)
Supplement: Supplementary file 2 — Protein extraction, digestion and labeling [file 41398_2020_1056_MOESM2_ESM.docx]

**Protein extraction, digestion and labelling**

Confluent cultures of hiPSC-NSC (5 days *in vitro*) or differentiated 3D neural aggregate cultures (14 days *in vitro*) were used. For sample preparation, cultivation media were aspirated and cultures were three-times rinsed with cold PBS (-Ca/-Mg), Cells were detached by Accutase and transferred to 1 ml conical tubes for centrifugation (1200 rpm, 5 min). After centrifugation, PBS was removed and cell pellets were homogenized using the lysis matrix D on FastPrep®-24 instrument (MP Biomedicals, OH) in lysis buffer (40-580 μl depending on size of sample, 50 mM triethylammonium bicarbonate (TEAB), 2% sodium dodecyl sulfate (SDS)) and 5 cycles 40 s each. The samples were centrifuged at maximum speed for 15 min, and supernatants transferred into a new vial. The protein concentration was determined using Pierce™ BCA Protein Assay (Thermo Scientific) and the Benchmark Plus microplate reader (BIO-RAD) with BSA solutions as standards. A representative reference was created containing equal amounts of the nine NSC and the eight neuron samples.

Aliquots containing 30 μg of each sample and the references were digested with trypsin using the filter-aided sample preparation (FASP) method (Wisniewski JR et. al. Nat Methods. 2009 May;6(5):359-62). Briefly, samples were reduced with 100 mM dithiothreitol at 60°C for 30 min, transferred to 30 kDa MWCO Pall Nanosep centrifugation filters (Sigma-Aldrich), washed repeatedly with 8 M urea and once with digestion buffer (1% sodium deoxycholate (SDC) in 50 mM TEAB) prior to alkylation with 10 mM methyl methanethiosulfonate in digestion buffer for 30 min. Digestion was performed in digestion buffer by addition of 0.3 µg Pierce MS grade Trypsin (Thermo Fisher Scientific) at 37°C and incubated overnight. An additional portion of trypsin was added and incubated for another two hours. Peptides were collected by centrifugation. Digested peptides were labelled using TMT 10-plex isobaric mass tagging reagents (Thermo Scientific) according to the manufacturer instructions and SDC was removed by acidification with 10% TFA.

The combined samples in each of the two TMT sets were pre-fractionated with basic reversed-phase chromatography (bRP-LC) using a Dionex Ultimate 3000 UPLC system (Thermo Fischer Scientific). Peptide separations were performed using a reversed-phase XBridge BEH C18 column (3.5 μm, 3.0x150 mm, Waters Corporation) and a linear gradient from 3% to 40% solvent B over 17 min followed by an increase to 100% B over 5 min. Solvent A was 10 mM ammonium formate buffer at pH 10.00 and solvent B was 90% acetonitrile, 10% 10 mM ammonium formate at pH 10.00. The 40 fractions were concatenated into 20 fractions, dried and reconstituted in 3% acetonitrile, 0.2% formic acid.

**NanoLC MS analysis and database search**

Each fraction was analysed on a Orbitrap Fusion Tribrid mass spectrometer interfaced with an Easy-nLC 1200 nanoflow liquid chromatography system (Thermo Fisher Scientific). Peptides were trapped on an Acclaim Pepmap 100 C18 trap column (100 μm x 2 cm, particle size 5 μm, Thermo Fischer Scientific) and separated on an in-house packed analytical column (75 μm x 300 mm, particle size 3 μm, Reprosil-Pur C18, Dr. Maisch) using a linear gradient from 5% to 35% B over 75 min followed by an increase to 100% B over 5 min and then 100% B for 5 min at a flow of 300 nL/min. Solvent A was 0.2% formic acid and solvent B was 80% acetonitrile in 0.2% formic acid. Precursor ion mass spectra were acquired at a resolution of 120,000 and MS/MS analysis was performed in a data-dependent multinotch mode where CID spectra of the most intense precursor ions were recorded in ion trap at collision energy setting of 35 for 3 s (‘top speed’ setting). Charge states 2 to 7 were selected for fragmentation, dynamic exclusion was set to 60 s and 10 ppm. MS^3^ spectra for reporter ion quantitation were recorded at a resolution of 50,000 with HCD fragmentation at collision energy of 65 using the synchronous precursor selection.

The data files for each TMT set were merged for identification and relative quantification using Proteome Discoverer version 2.2 (Thermo Fisher Scientific). The database search was performed using the human Swissprot Database (Jan 2019) using Mascot 2.5.1 (Matrix Science) as a search engine with precursor mass tolerance of 5 ppm and fragment mass tolerance of 0.6 Da. Tryptic peptides were accepted with zero missed cleavage, variable modifications of methionine oxidation and fixed cysteine alkylation, TMT-label modifications of N-terminal and lysine were selected. Percolator was used for the validation of identified proteins and the quantified proteins were filtered at 1% FDR and grouped by sharing the same sequences to minimize redundancy. Reporter ion intensities were quantified in MS3 spectra at 0.003 Da mass tolerance using the S/N values as abundances and normalized on the total protein abundance within the Proteome Discoverer 2.2 workflow. Only peptides unique for a given protein were considered for quantification of the protein.
