# Supplementary figures and images for: Identification of candidate genetic variants and altered protein expression in neural stem and mature neural cells support altered microtubule function to be an essential component in bipolar disorder
Source: Transl Psychiatry. 2020 Nov 9;10:390. doi: 10.1038/s41398-020-01056-1 (PMC7652854; doi:10.1038/s41398-020-01056-1)

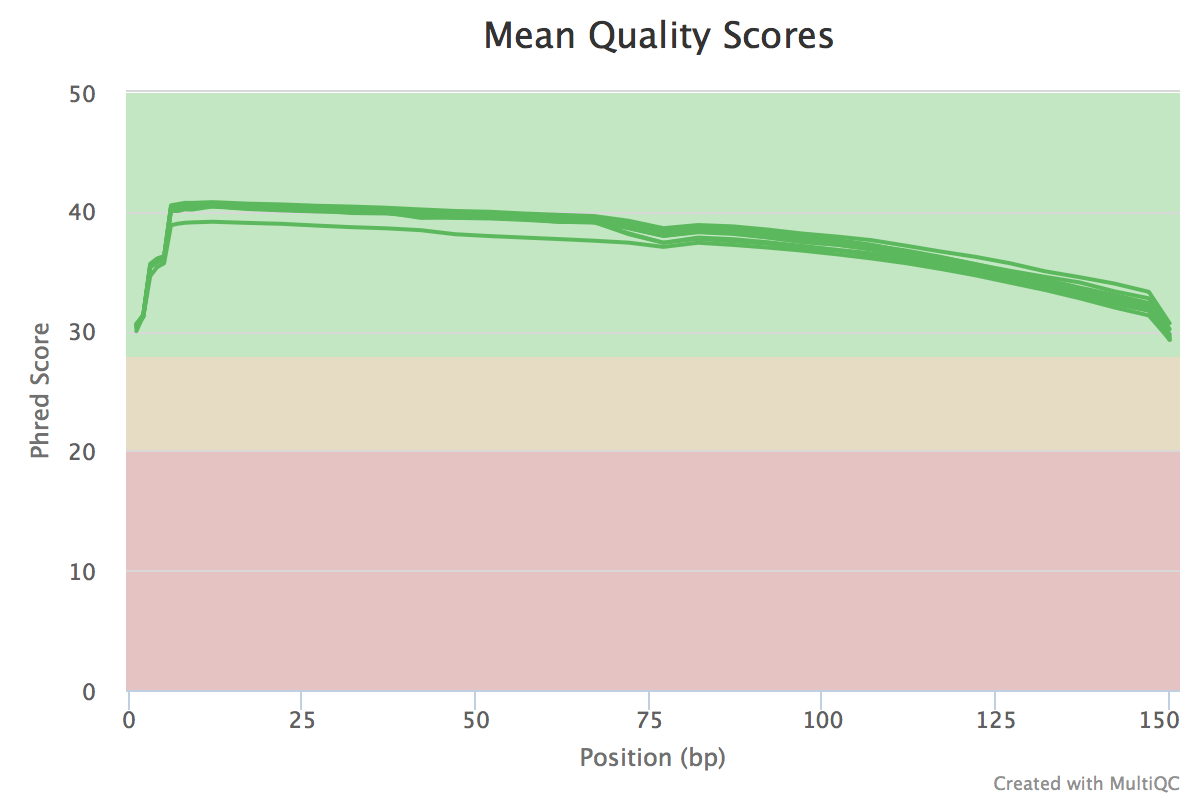

Supplement: Supplementary file 3 — Figure S1 [file 41398_2020_1056_MOESM3_ESM.png]
